# Heritability of Working in a Creative Profession

**DOI:** 10.1007/s10519-016-9832-0

**Published:** 2016-12-15

**Authors:** Mark Patrick Roeling, Gonneke Willemsen, Dorret I. Boomsma

**Affiliations:** 1 Department of Computer Science, University of Oxford, Robert Hooke Building, Parks Road, Oxford, OX1 3QD UK; 2 Department of Biological Psychology, Vrije Universiteit Amsterdam, Amsterdam, The Netherlands

**Keywords:** Creativity, Twin study, Talent, Working, Profession, Heritability

## Abstract

Creativity is the tendency to generate or recognize ideas, alternatives, or possibilities. Following a study on the genetic contribution to working in a creative profession, based on polygenic score analysis, we report the total heritability of this trait in a large sample of adult twins and their siblings registered with the Netherlands Twin Register. Data from 6755 twins and 1817 siblings were analyzed using genetic structural equation modeling. Working in a creative profession is relatively rare in our sample (2.6% of twins and 3.2% of siblings). Twin correlations (identical 0.68 and fraternal 0.40) commended a model with additive genetic factors (full model estimate 0.56), shared (full model estimate 0.12), and unique environmental factors (full model estimate 0.32). Genetic model fitting resulted in a best-fitting model existing of additive genetic factors and unique environmental factors, resulting in a heritability of 0.70.

## Introduction

Creativity is the tendency to generate or recognize ideas, alternatives, or possibilities that may be useful in solving problems, communicating with others, and entertaining ourselves and others (Franken 2006). This broad array of discrete abilities has a strong cognitive component (Weisberg 1992) and creativity correlates with intelligence and cognitive performance (Guilford 1967; Penke 2006).

There is ample evidence for the influence of genetic factors but heritability estimates are diverse. Ten early twin studies were summarized in a review presenting average correlations of 0.61 for MZ twins and 0.50 for DZ twins (Nichols 1978), resulting in a rough heritability estimate of 25% (and 38% shared environmental factors; Penke 2006). A subsequent study into perceptual and esthetic abilities (Barron and Parisi 1976) also argued in favor of hereditary influences in creativity. Generally, studies in adolescents provide lower heritability estimates of creativity-related traits. One twin study in adolescents (13–19 years) examined 11 creative ability measures (e.g. recognizing obscure figures or create story plot titles) and observed only three scales with significant MZ-DZ differences (Reznikoff et al. 1973). A Russian twin study estimated a heritability of 0.44 in creative thinking (Grigorenko et al. 1992). Compared to the aforementioned studies in adolescents, heritability estimates are slightly higher in adults for traits as creative personality (50–54%; Bouchard et al. 1993; Velazquez et al. 2015), drawing (38–47%; Velazquez et al. 2015), arts (60%; Vinkhuyzen et al. 2009), creative writing (83%; Vinkhuyzen et al. 2009), creative achievement (61%; Piffer and Hur 2014), perceived (62%) and figural (26%) creativity (Kandler et al. 2016). Variability in creativity over age has been interpreted as indicative for cognitive maturity, with children first becoming proficient learners while the ability to think creatively evolves throughout development (Fogarty et al. 2015).

Another aspect of creativity is the phenotypic and genotypic overlap with traits such as openness, extraversion, and intelligence (Canter 1973; Kandler et al. 2016; Penke 2006), which extends to extreme expressions of behavior (Carson 2011) illustrated by the co-occurrence of creativity with attention problems (Mayseless et al. 2013), schizophrenia and bipolar disorder (Power et al. 2015). The latter study linked genetic variants underlying schizophrenia and bipolar disorder to creativity, explaining 0.24 and 0.26% of the variance of creativity, respectively. Power et al. (2015) analyzed creativity as working in a creative profession, but did not report the overall heritability of this trait. Therefore, we now use data from 6755 twins and 1817 siblings aged ≥21 years registered with the NTR (Willemsen et al. 2013), to estimate the total heritability of working in a creative profession. Extending the classical twin design of mono- and dizygotic twins with their siblings gives larger statistical power to estimate both shared environmental and non-additive genetic effects (Posthuma and Boomsma 2000).

## Methods

### Participants

This study is part of an ongoing study on health, lifestyle and personality in twins and their family members registered in the NTR. Every two to three years, registered families receive surveys on health and lifestyle and the present study uses from the seventh and eight surveys respectively collecting data in 2004–2008 and 2009–2012 (described in Willemsen et al. 2013). Informed consent was obtained from all individual participants included in the study. Zygosity was determined either through genotyping or from self- and parental report answers to survey questions on physical resemblance or confusion of the twins by other family members and peers. DNA and survey zygosity agreement reached more than 96%.

Data were available from 8802 participants (N_TWINS_ = 6942, N_SIBLINGS_ = 1860). We removed individuals with age unknown (N = 6) or age below 20 years (N = 3) and families including only one twin (N = 95), multiple twins from a different pair (N = 31), or twins with missing zygosity (N = 27). A maximum of two brothers and two sisters were included in the analyses, remaining siblings were excluded (N = 68). In families with triplets, we selected two random twins, and if dizygotic, the remaining twin was used as a sibling. In families with multiple twin pairs, we selected the first twin pair and used the other twin pairs to extract one random twin as sibling. This resulted in a total sample of 6755 twins and 1817 siblings from 4734 families, including 999 monozygotic males (MZM), 541 dizygotic males (DZM), 2585 monozygotic females (MZF), 1249 dizygotic females (DZF), 1381 DZ opposite-sex pairs, with 679 brothers and 1138 sisters. Table 1 shows the complete family configuration of the sample. There were 2427 families in which both members of a twin pair completed the questionnaire, 1901 families in which only one member of the twin pair completed the questionnaire and 406 families in which only non-twin siblings completed the questionnaire (added to DZMales). The mean age of the twins was 38.42 years [standard deviation (SD) 11.98, range 20–90 years] and the mean age for their siblings was 41.26 years (SD 12.18, range 20–90 years).

**Table 1:** Family structures in dataset

	Families yielding					
	No siblings	1 sibling	2 siblings	3 siblings	4 siblings	Total
MZM						
Families yielding a twin pair	258	81	14	10	1	364
Families yielding a single twin	233	26	11	0	1	271
DZM						
Families yielding a twin pair	108	44	8	3	1	164
Families yielding a single twin	165	36	10	2	0	213
MZF						
Families yielding a twin pair	777	220	53	9	5	1064
Families yielding a single twin	400	46	10	1	0	457
DZF						
Families yielding a twin pair	300	95	39	8	0	442
Families yielding a single twin	314	43	6	2	0	365
DOS						
Families yielding a twin pair	260	101	24	6	2	393
Families yielding a single twin	498	75	16	6	0	595
Families yielding no twins	–	335	62	8	1	406
Total	3315	1102	253	55	11	4734

Family structures in dataset

*MZM* monozygotic males, *DZM* dizygotic males, *MZF* monozygotic females, *DZF* dizygotic females, *DOS* DZ opposite-sex

### Measures

Following an earlier study (Power et al. 2015), creativity (being in a creative profession) scoring relied on surveys including detailed questions about the participants’ occupations. Using a detailed description, individuals were asked to report their profession. We then classified these professions on being creative or not. Creative professionals were defined as those having positions in the fields of dance, film, music, theatre, visual arts, or writing. We did not differentiate whether, within these categories, persons were more or less creative. When persons were not working or reported to be a housewife at the moment of data completion, they were asked for their past occupation and this was used to assess whether they had a creative profession. When a person indicated to be a housewife and had not had another profession in the past, this was coded as not involved in a creative profession. In the case of full-time or part-time education, creative profession was regarded missing.

### Statistical analyses

Descriptive analyses were performed in SPSS v.20. Genetic structural equation model analyses were conducted in Mx (Mx: statistical modeling; Neale 2006). Given the categorical nature of the data (yes/no) we fitted a ‘liability’ model (Falconer 1965), in which the categorical variable was assumed to reflect an imprecise measurement of an underlying normal distribution of liability. Being a theoretical construct, the liability’s scale needs to be defined. In general, the liability is assumed to be standard normally distributed with zero mean and unit variance. The threshold acts as a reference of incidence of the different categories in the population (Falconer and Mackay 2005).

We fitted different sets of models to the raw ordinal data using maximum likelihood estimation. In the fully saturated model, thresholds were allowed to vary as a function of sex (0 for male and 1 for female) and twin/sib status. Specifying separate thresholds for twins and siblings allows to investigate sibling effects (a specific kind of genotype-environment autocorrelation), which can occur because the genotype of one sibling or twin is genetically correlated with the phenotype of the other sibling which is providing part of the environment, potentially resulting in different thresholds between siblings (Neale and Maes 2002). Age (standardized and sex specific coefficients) was modelled as a covariate on the threshold to account for any remaining variability in incidence of creativity as a function of age, expecting similar effects of the age β coefficient between zygosity groups. Tetrachoric correlations were estimated for the continuous liability distribution, with a total of eight correlations for MZMales, DZMales, MZFemales, DZFemales, DZ opposite-sex pairs, brother–brother, sister–sister, and brother–sister) to be estimated. In total, the saturated model comprised of 16 free parameters: one threshold for male twins and one threshold for female twins, one threshold for brothers and one for sisters, two fixed effects, i.e., covariates coefficients (age and sex) for males and two coefficients for females, and eight correlations estimating the familial resemblance. In a series of nested models we tested constraints to test the significance of different parameters and derive the most parsimonious model. The fit of submodels was evaluated with Log-likelihood ratio testing, which involves subtracting the negative log-likelihood (−2LL) for the more general model from the −2LL of the more restricted model. This gives a *χ* ^2^ test with the degrees of freedom (*df*) equal to the difference in the number of estimated parameters in the two models. A significant *χ* ^2^ (*p* < 0.05) indicates that the constrained model fits significantly worse than the previous model. As a result, the previous model is kept as the most parsimonious model, to which a new model can be compared. Thus, those models that are the most parsimonious and efficient representations of the data are selected.

### Genetic analyses

From the difference in genetic relatedness in MZ and DZ twins, who share respectively 100 and 50% (on average) of their segregating genes, the amount of variance can be estimated and ascribed to genetic and environmental factors (Boomsma et al. 2002). A higher MZ correlation compared to the DZ correlation is indicative of genetic influences. If MZ and DZ correlations are similar, genetic effects are not suggested.

Quantitative genetic modeling is based on the fact that the phenotypic variance is a function of genetic, shared, and unique environmental variance. The expectation for the phenotypic variance may be written as: V_(P)_ = V_(A)_ + V_(D)_ + V_(C)_ + V_(E)_. Genetic variance can be additive (A), indicating that the effects of multiple alleles are additive, or nonadditive (dominance, D) meaning that alleles at a particular locus interact. When the DZ correlation is more than half the MZ correlation, there is evidence for environmental effects shared by twins from the same family (C) and when the DZ correlation is less than half the MZ correlation, there is evidence for non-additive genetic effects. Broad-sense heritability (h^2^) is the proportion of phenotypic variance that is attributable to genotypic variance (h^2^ = (V_(A)_ + V_(D)_)/V_(P)_); narrow-sense heritability is the proportion of variation explained by additive genetic factors (h_n_^2^ = V_(A)_/V_(P)_). A classical twin design only provides information to model either an ACE model or an ADE model, but adding data from siblings of twins provides more information and statistical power to distinguish between additive and dominant genetic factors. The additive genetic variance is perfectly correlated in MZ twins, whereas for DZ twins and siblings the cross-twin/cross-sib correlation between the A factors is 0.5. Again, the significance of genetic parameters was tested by comparing submodels against a more general model, using log-likelihood ratio testing.

## Results

We observed a low frequency of working in a creative profession with 175 (2.6%) of 6755 twins and 58 (3.2%) of 1817 siblings reporting positive (see Table 2). For each family, we indicated the number of individuals within the family, restricted to twins, siblings and parents. From the 8572 individuals (twins with ≤4 siblings) included in the statistical analyses, no data on additional family members were available in 1331 persons. In the remaining 7241 individuals with data from family members: 4.5% had 1 or more family members in a creative profession. We then split the data according to creative profession of the person itself. Of the persons who were, themselves, not in a creative profession (N = 7042), 3.9% had family members in a creative profession. In the persons who were in a creative profession (N = 199), 26.1% had family members in a creative profession.

**Table 2:** Reported creative professions

	N (%) for first profession	N (%) for second profession
Architecture	18 (7.6)	–
Art teacher	15 (6.4)	7 (21.9)
Art therapy	1 (0.4)	–
Artisan	16 (3.8)	6 (18.8)
Cinematography	20 (8.5)	3 (9.4)
Creative director	13 (5.5)	2 (6.3)
Creative writer	5 (2.1)	–
Curator	2 (0.8)	–
Dance teacher	4 (1.7)	1 (3.1)
Fashion design	2 (0.8)	–
Flower design	15 (6.4)	–
Game design	1 (0.4)	–
Graphical design	41 (17.4)	–
Illustrator	2 (0.8)	–
Interior design	18 (7.6)	–
Landscape architect	3 (1.3)	–
Music teacher	9 (3.8)	–
Musician	3 (1.3)	–
Photography	2 (0.8)	1 (3.1)
Reporter	19 (8.1)	–
Set decoration	3 (1.3)	–
Singer	1 (0.4)	–
Theatre artist	7 (3.0)	1 (3.1)
Theatre teacher	5 (2.1)	1 (3.1)
Web design	7 (3.0)	–
Writer	4 (1.7)	9 (28.1)
Costume maker	–	1 (3.1)
Total	236 (100)	32 (100)

The Artisan category includes individuals working as a fine artist (painter, art drawing, and ceramics artist). Three individuals reported two creative professions as first profession

Between twins, there was no evidence for birth-order effects ($\chi_{(1)}^{2}$ = 0.010, *p* = 0.920). Of the 4734 families, 4523 (95.5%) had no family members who are who are working, or worked, in a creative profession. Table 3 presents the results of the tests in the saturated model. Creativity scores, measured as thresholds, were not significantly different for male twins and brothers nor for female twins and sisters ($\chi_{(1)}^{2}$ = 1.759, *p* = 0.415) suggesting the absence of sibling effects. Thresholds could also be constrained across gender ($\chi_{(1)}^{2}$ = 0.032, *p* = 0.857). In females, there was a significant positive age effect ($\chi_{(1)}^{2}$ = 12.309, *p* ≤ 0.001) indicating that older females more frequently report to work, or to have worked, in a creative profession. Phenotypic correlations between twins were 0.54 in MZMales and 0.69 in MZFemales versus 0.47 in DZMales, 0.25 in DZFemales and 0.31 in DZ-opposite sex pairs. Sibling correlations were 0.54 between brothers, 0.31 between sisters, and 0.51 between sisters and brothers. Constraining the phenotypic correlations between twins and siblings (e.g., brother–brother correlation to DZMales) and between males and females did not result in a significant deterioration of the model fit, commending a model where the heritability is equal for both gender groups. After constraining the correlations, the constrained MZ (MZMales and MZFemales) correlation was 0.68 and the constrained DZ (DZ twins and siblings) correlation was 0.40, suggesting the influence of shared environmental factors (C) and providing evidence for an ACE model.

**Table 3:** Model fit results for the saturated model

Model	Test	Versus	−2LL	*df*	*χ* ^2^	Δ*df*	*p*
0	Full model	–	2115.081	8572			
1	Thresholds male twins = brothers Threshold female twins = sisters	0	2116.840	8574	1.759	2	0.415
2	Thresholds males = females	1	2116.872	8575	0.032	1	0.857
3	Age effects males = females	2	2122.821	8576	5.949	1	0.015
3a	Age effects males = 0	2	2116.962	8576	0.090	1	0.764
3b	Age effects females = 0	2	2129.181	8576	12.309	1	<0.001
4	Sex effects males = females	3a	2117.011	8577	0.049	1	0.825
4a	Sex effects males and females = 0	4	2117.107	8578	0.096	1	0.757
5	DZ twin correlations = sibling correlations	4a	2117.860	8581	0.753	3	0.861
6	Male correlations = female correlations	5	2118.288	8583	0.428	2	0.807
**7**	Same sex DZ/sibling correlations = opposite sex DZ/sibling correlations	**6**	**2119.074**	**8584**	**0.786**	**1**	**0.375**

Model fit results for the saturated model. The best fitting model had four free parameters (one threshold, MZ correlation, DZ correlation, and age coefficient females). The best fitting model is printed in bold

−*2LL* −2 log likelihood, *df* degrees of freedom, *p p* value, *DZ* dizygotic

The full model estimated A to be 0.56 (95% CI 0.11–0.80), C to be 0.12 (95% CI 0–0.45) and E to be 0.32 (95% CI 0.19–0.50). Dropping the shared environmental factor from the ACE model did not significantly worsen the model fit ($\chi_{(1)}^{2}$ = 0.478, *p* = 0.489), whereas removal of the additive genetic component did ($\chi_{(1)}^{2}$ = 5.997, *p* = 0.014). Therefore, the best fitting model is an AE model with a heritability estimate of 0.70.

## Discussion

This study estimated the heritability of working in a creative profession in a large sample of Dutch adult twins and their siblings. The best fitting model yielded a heritability estimate of 70% with the remaining variance explained by unique environmental factors. Our finding confirms earlier smaller studies in adults (Bouchard et al. 1993; Kandler et al. 2016; Penke 2006; Piffer and Hur 2014; Vinkhuyzen et al. 2009) presenting evidence for significant genetic influences underlying creativity. Outcomes of this study are also in line with twin studies investigating personality traits where the majority of studies show a heritability of 0.49–0.57 and low to zero shared environmental influences (Bouchard and McGue 2003). The insignificant prevalence differences and similar heritability estimates between sexes conforms to earlier work (Penke 2006; Piffer and Hur 2014) and adds evidence to a field where studies that report higher creativity scores in females are counterbalanced (in number) by studies where males score higher (Baer and Kaufman 2006).

This study is limited in using only self-report data on profession that is categorized by us as creative or not creative, and in the low prevalence of working in a creative profession. Possibly, the focus on working in a creative profession provides an underestimate of creativity in general, as participants can still be creative in their own time (e.g., during leisure) which is not captured by this study. The combination of subtle influences of C (ACE model estimate = 0.12) and the use of a threshold model in an unbalanced dataset reduces the power of our method. The inclusion of (at least one) additional sibling(s) is a proven strategy to increase the power to accurately estimate variance components, especially common environmental factors (Posthuma and Boomsma 2000). From Neale et al. (1994)we deduce that with our prevalence (between 0.01 and 0.05) a sample size at least 100,000 twins would be needed to detect the AE model under a true C of 0.12. Despite this being one of the largest twin studies focusing on creativity to date, there may be much to learn about shared environmental effects that contribute to the decision to pursue a creative carreer with more powerful samples or methods. From our work and others, the substantial heritability estimate for the phenotype indicates that multiple DNA variants, in addition to those overlapping with schizophrenia and bipolar disorder are likely to be found in the future (Illustration 1).

**Illustration 1:**
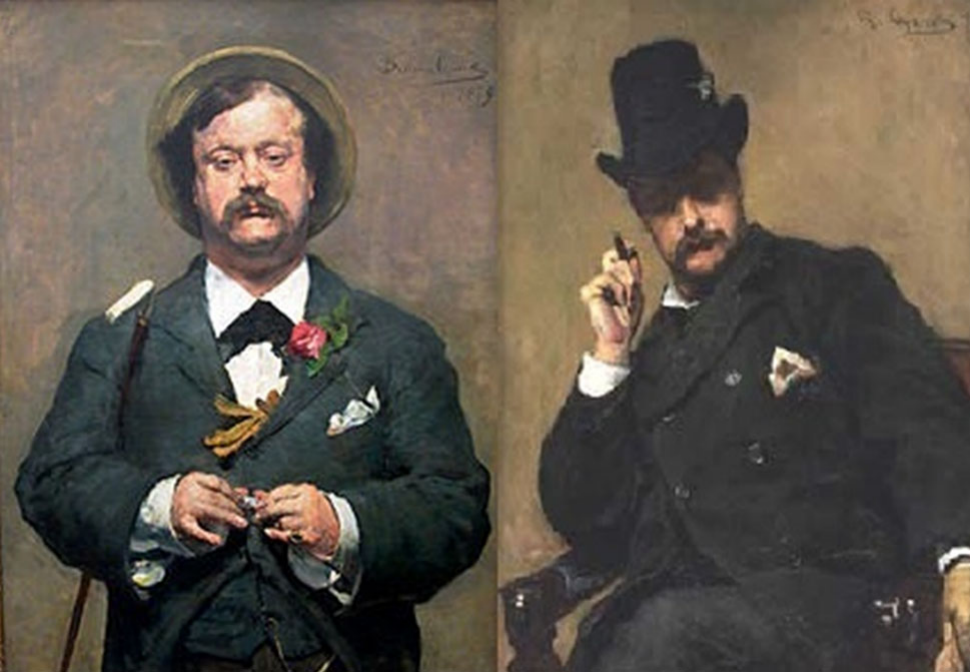
MZ twins are more often concordant for creativity, as illustrated by these portraits where Dutch MZ twins David and Pieter Oyens (successful nineteenth century painters) painted each other
